# The 100 most influential manuscripts in andrology: a bibliometric analysis

**DOI:** 10.1186/s12610-018-0080-4

**Published:** 2018-12-12

**Authors:** Nicholas Bullock, Thomas Ellul, Adam Bennett, Martin Steggall, Gareth Brown

**Affiliations:** 1 Division of Cancer and Genetics, Cardiff University School of Medicine, Heath Park, Cardiff, CF14 4XN UK; 2 Department of Urology, Cwm Taf University Health Board, Royal Glamorgan Hospital, Llantrisant, CF72 8XR UK; 3 Faculty of Life Sciences and Education, University of South Wales, Pontypridd, CF37 4BD UK

**Keywords:** Andrology, Influential, Citation rank, Bibliometric analysis

## Abstract

**Background:**

As the specialty of Andrology expands it is important to establish the most important studies that have shaped, and continue to shape, current research and clinical practice. Bibliometric analysis involving a citation rank list is an established means by which to identify the published material within a given field that has greatest intellectual influence. This bibliometric analysis sought to identify the 100 most influential manuscripts in Andrology, as well as the key research themes that have shaped contemporary understanding and management of andrological conditions.

**Methods:**

The Thompson Reuters Web of Science citation indexing database was interrogated using a number of search terms chosen to reflect the full spectrum of andrological practice. Results were ranked according to citation number and further analysed according to subject, first and senior author, journal, year of publication, institution and country of origin.

**Results:**

The Web of Science search returned a total of 24,128 manuscripts. Citation number of the top 100 articles ranged from 2819 to 218 (median 320). The most cited manuscript (by Feldman et al., The Journal of Urology 1994; 2819 citations) reported the prevalence and risk factors for erectile dysfunction (ED) in the Massachusetts Male Ageing Study. The Journal of Urology published the highest number of manuscripts (*n* = 11), followed by the New England Journal of Medicine (*n* = 10). The most common theme represented within the top 100 manuscripts was erectile dysfunction (*n* = 46), followed jointly by hypogonadism and male factor infertility (*n* = 24 respectively).

**Conclusion:**

Erectile dysfunction should be considered the most widely researched, published and cited field within andrological practice. This study provides a list of the most influential manuscripts in andrology and serves as a reference of what comprises a ‘highly citable’ paper for both researchers and clinicians.

## Résumé

**Contexte:**

Etant donné que la spécialité Andrologie se développe, il est important de dresser un inventaire des études les plus importantes qui ont façonné, et qui continuent à façonner, la recherche actuelle et la pratique clinique. L’analyse bibliométrique, qui inclue une liste de rang de citations, est un moyen bien établi pour identifier le matériel publié, dans un champ donné, qui a la plus grande influence intellectuelle. La présente analyse bibliométrique cherche à identifier les 100 manuscrits les plus influents en Andrologie, ainsi que les principaux thèmes de recherche qui ont façonné la compréhension et la prise en charge contemporaines des situations andrologiques.

**Méthodes:**

La base de données d’indexation des citations du Web of Science de Thompson Reuters a été interrogée en utilisant un nombre de termes de recherche choisis pour refléter l’éventail complet de la pratique andrologique. Les résultats ont été classés selon leur nombre de citations, puis ensuite analysés en fonction du sujet, des premier et dernier auteurs, du journal, de l’année de publication, de l’institution et du pays d’origine.

**Résultats:**

La recherche sur le Web of Science a rapporté un total de 24 128 manuscrits. Le nombre de citations des 100 premiers articles va de 2 819 à 218 (médiane 320). L’article le plus cité (de Feldman et al., The Journal of Urology 1994 ; 2 819 citations) rapportait la prévalence et les facteurs de risque de la dysfonction érectile (DE) dans l’étude du Massachusetts menée chez l’homme vieillissant. The Journal of Urology a publié le plus grand nombre de manuscrits (n=11), suivi du New England Journal of Medicine (n=10). Le thème le plus souvent représenté dans les manuscrits du top 100 était la dysfonction érectile (n=46), suivi conjointement par l’hypogonadisme et l’infertilité d’origine masculine (respectivement n=24).

**Conclusion:**

La dysfonction érectile devrait être considérée comme le champ de la pratique andrologique qui a le plus largement fait l’objet de recherches, de publications et de citations. La présente étude fournit une liste des manuscrits les plus influents en andrologie, et constitue une référence sur ce que signifie un article ‘fortement cité’ à la fois pour les chercheurs et pour les cliniciens.

## Background

Andrology is the medical specialty that focuses on many aspects of male health, comprising a wide range of conditions of the male reproductive system, as well as urological pathologies that are specific to men. Although the clinical science had been studied for many years, it was not until after the introduction of the term ‘andrology’ in 1951 that scientists and clinicians from a diverse range of backgrounds began to refer to themselves as ‘andrologists’ [1]. Since then the discipline has continued to evolve, with the introduction of national and international societies, recognised and accredited training courses and a number of high quality dedicated journals. In the present day, andrologists are involved in the management of a wide spectrum of diseases ranging from male factor infertility through to hypogonadism and penile cancer. As the specialty grows and the body of literature focusing on andrological topics expands it is important to establish the most important and influential manuscripts that have shaped, and continue to shape, current research and clinical practice.

The generation of a citation rank list is one method of identifying the published material within a given field that has greatest intellectual influence [2]. A citation refers to the referencing of an article by another peer-reviewed publication. It is therefore probable that articles which have the greatest impact on the scientific and clinical communities are cited many times more than those which have had little impact. Citation analysis is the process of ranking the most frequently cited articles in order to produce a citation rank list. In addition, citation numbers can be used to rank journals through calculation of their ‘impact factor.’ This is a measure of the average number of citations a manuscript published in a particular journal received during a specific timeframe and is often used a surrogate marker for journal quality.

A number of clinical disciplines have employed citation analysis to determine the most influential articles in their field. These include entire specialties such as general surgery [3], plastic surgery [4] and orthopaedic surgery [5], as well as subspecialties such as laparoscopic and emergency abdominal surgery [6, 7]. Whilst citation analysis has been conducted within urology as a whole [8, 9], and more specifically for male factor infertility [10], no study to date has been undertaken to determine the most influential manuscripts in andrology. This bibliometric analysis therefore aimed to identify the most influential articles in the field, as well as key research themes that have been instrumental in developing our contemporary understanding and management of andrological conditions.

## Methods

The Thompson Reuters Web of Science citation indexing database was interrogated using the method previously described by Ellul et al. [6]. As andrology is a broad subspecialty that draws from a range of other disciplines, it is probable that influential articles pertaining to relevant topics have been published in a wide array of journals, not just those specific to urology and sexual medicine. A number of title search terms were therefore selected and combined to ensure all relevant manuscripts were identified, as follows: ‘andrology’, ‘male infertility’, ‘erectile dysfunction’, ‘impotence’, ‘penile deformity’, ‘penile curvature’, ‘peyronie’s disease’, ‘priapism’, ‘penile fracture’, ‘ejaculatory disorder’, ‘male sexual dysfunction’, ‘hypogonadism’, ‘penile cancer’, ‘squamous cell carcinoma’ and ‘penis’. Truncation using the asterisk function (*) was utilised for particular words with multiple relevant variations, for example the term ‘peni*’ was used to capture both ‘penis’ and ‘penile’. These search terms were chosen to reflect the core topics published in the European Academy of Andrology-European Society of Andrological Urology Joint Educational Curriculum for Clinical Andrology Training in Europe [11].

The search was conducted on 5th June 2018 and included all manuscripts published in the English language from 1900 onwards. Results were subsequently ranked by citation number. Final interrogation of the database was independently performed by two assessors (NB and TE). The 100 most cited articles were further evaluated according to subject, first and senior author, journal, year of publication, institution and country of origin. The 2016 impact factor of each journal was also identified from the Journal Citation Reports dataset [12]. In order to adjust for older articles accruing a higher number of citations over time the citation rate was calculated by dividing the number of citations by the number of years since publication. Articles were excluded if published prior to 1900, in languages other than English and/or if, after independent assessment by two researchers, it was agreed the main focus was not directly relevant to the field andrology. In cases of disagreement on the suitability for inclusion, the manuscript in question was discussed by both assessors and a consensus decision reached.

## Results

The Web of Science search returned a total of 24,128 manuscripts. Table 1 lists the 100 most cited articles as ranked by citation number, following application of exclusion criteria. Where two articles had equal numbers of citations, further stratification was based on citation rate. The most cited article was that by Feldman et al. describing the prevalence of and risk factors for erectile dysfunction (ED) in the Massachusetts Male Ageing Study, published in The Journal of Urology in 1994 and has been cited 2819 times [13].

**Table 1:** The 100 most cited manuscripts in Andrology

Rank	Manuscript (first author, title, journal and year)	Citations
1	Feldman HA. Impotence and its medical and psychosocial correlates - results of the Massachusetts male aging study. Journal of Urology 1994.	2819
2	Rosen RC. The international index of erectile function (IIEF): A multidimensional scale for assessment of erectile dysfunction. Urology 1997.	2480
3	Goldstein I. Oral sildenafil in the treatment of erectile dysfunction. New England Journal of Medicine 1998.	1515
4	Rosen RC. Development and evaluation of an abridged, 5-item version of the International Index of Erectile Function (IIEF-5) as a diagnostic tool for erectile dysfunction. International Journal of Impotence Research 1999.	1325
5	Droller MJ. Impotence: NIH consensus development panel on impotence. Journal of the American Medical Association 1993.	1270
6	de Roux N. Hypogonadotropic hypogonadism due to loss of function of the KiSS1-derived peptide receptor GPR54. Proceedings of the National Academy of Sciences of the United States of America 2003.	1237
7	Walsh PC. Impotence following radical prostatectomy - insight into etiology and prevention. Journal of Urology 1982.	1044
8	Lue TF. Drug therapy: Erectile dysfunction. New England Journal of Medicine 2000.	793
9	Rosen R. Lower urinary tract symptoms and male sexual dysfunction: The multinational survey of the aging male (MSAM-7). European Urology 2003.	675
10	Evenson DP. Sperm chromatin structure assay: Its clinical use for detecting sperm DNA fragmentation in male infertility and comparisons with other techniques. Journal of Andrology 2002.	610
11	Eddy EM. Targeted disruption of the estrogen receptor gene in male mice causes alteration of spermatogenesis and infertility. Endocrinology 1996.	607
12	Johannes CB. Incidence of erectile dysfunction in men 40 to 69 years old: Longitudinal results from the Massachusetts male aging study. Journal of Urology 2000.	558
13	Aytac IA. The likely worldwide increase in erectile dysfunction between 1995 and 2025 and some possible policy consequences. British Journal of Urology International 1999.	553
14	Muscatelli F. Mutations in the dax-1 gene give rise to both x-linked adrenal hypoplasia congenita and hypogonadotropic hypogonadism. Nature 1994.	536
15	Wu FCW. Identification of Late-Onset Hypogonadism in Middle-Aged and Elderly Men. New England Journal of Medicine 2010.	524
16	Katznelson L. Increase in bone density and lean body mass during testosterone administration in men with acquired hypogonadism. Journal of Clinical Endocrinology & Metabolism 1996.	516
17	Sharma RK. Role of reactive oxygen species in male infertility. Urology 1996.	516
18	Braun M. Epidemiology of erectile dysfunction: results of the ‘Cologne Male Survey’. International Journal of Impotence Research 2000.	511
19	Mason AJ. A deletion truncating the gonadotropin-releasing-hormone gene is responsible for hypogonadism in the hpg mouse. Science 1986.	492
20	Krane RJ. Impotence. New England Journal of Medicine 1989.	482
21	Thompson IM. Erectile dysfunction and subsequent cardiovascular disease. Journal of the American Medical Association 2005.	*481*
22	Tremellen K. Oxidative stress and male infertility-a clinical perspective. Human Reproduction Update 2008.	479
23	Lue TF. Physiology of erection and pharmacological management of impotence. Journal of Urology 1987.	458
24	Cattanach BM. Gonadotrophin-releasing hormone deficiency in a mutant mouse with hypogonadism. Nature 1977.	453
25	de Tejada IS. Impaired neurogenic and endothelium-mediated relaxation of penile smooth-muscle from diabetic men with impotence. New England Journal of Medicine 1989.	438
26	Esposito K. Effect of lifestyle changes on erectile dysfunction in obese men - A randomized controlled trial. Journal of the American Medical Association 2004.	434
27	Feldman HA. Erectile dysfunction and coronary risk factors: Prospective results from the Massachusetts Male Aging Study. Preventive Medicine 2000.	431
28	Boolell M. Sildenafil, a novel effective oral therapy for male erectile dysfunction. British Journal of Urology 1996.	423
29	Terrett NK. Sildenafil (VIAGRA), a potent and selective inhibitor of type 5 cGMP phosphodiesterase with utility for the treatment of male erectile dysfunction. Bioorganic & Medicinal Chemistry Letters 1996.	421
30	Thorner MO. Long-term treatment of galactorrhea and hypogonadism with bromocriptine. British Medical Journal 1974.	421
31	Hatzimouratidis K. Guidelines on Male Sexual Dysfunction: Erectile Dysfunction and Premature Ejaculation. European Urology 2010.	420
32	Dubin L. Etiologic factors in 1294 consecutive cases of male infertility. Fertility and Sterility 1971.	418
33	Rendell MS. Sildenafil for treatment of erectile dysfunction in men with diabetes - A randomized controlled trial. Journal of the American Medical Association 1999.	417
34	Tut TG. Long polyglutamine tracts in the androgen receptor are associated with reduced trans-activation, impaired sperm production, and male infertility. Journal of Clinical Endocrinology & Metabolism 1997.	416
35	Carter JN. Prolactin-secreting tumors and hypogonadism in 22 men. New England Journal of Medicine 1978.	414
36	Whorton D. Infertility in male pesticide workers. Lancet 1977.	405
37	Topaloglu AK. TAC3 and TACR3 mutations in familial hypogonadotropic hypogonadism reveal a key role for Neurokinin B in the central control of reproduction. Nature Genetics 2009.	395
38	Dix DJ. Targeted gene disruption of Hsp70–2 results in failed meiosis, germ cell apoptosis, and male infertility. Proceedings of the National Academy of Sciences of the United States of America 1996.	393
39	Agarwal A. Role of sperm chromatin abnormalities and DNA damage in male infertility. Human Reproduction Update 2003.	389
40	Brindley GS. Cavernosal alpha-blockade - a new technique for investigating and treating erectile impotence. British Journal of Psychiatry 1983.	374
41	Padma-Nathan H. Treatment of men with erectile dysfunction with transurethral alprostadil. New England Journal of Medicine 1997.	354
42	Morales A. Clinical safety of oral sildenafil citrate (VIAGRA) in the treatment of erectile dysfunction. International Journal of Impotence Research 1998.	351
43	Virag R. Is impotence an arterial disorder - a study of arterial risk-factors in 440 impotent men. Lancet 1985.	349
44	Araujo AB. The relationship between depressive symptoms and male erectile dysfunction: Cross-sectional results from the Massachusetts Male Aging Study. Psychosomatic Medicine 1998.	345
45	Montorsi F. Erectile dysfunction prevalence, time of onset and association with risk factors in 300 consecutive patients with acute chest pain and angiographically documented coronary artery disease. European Urology 2003.	340
46	Mulligan T. Prevalence of hypogonadism in males aged at least 45 years: the HIM study. International Journal of Clinical Practice 2006.	331
47	Oliva R. Protamines and male infertility. Human Reproduction Update 2006.	330
48	Brock GB. Efficacy and safety of tadalafil for the treatment of erectile dysfunction: Results of integrated analyses. Journal of Urology 2002.	330
49	Dhindsa S. Frequent occurrence of hypogonadotropic hypogonadism in type 2 diabetes. Journal of Clinical Endocrinology & Metabolism 2004.	327
50	Roth JC. FSH and LH response to luteinizing hormone-releasing factor in prepubertal and pubertal children, adult males and patients with hypogonadotropic and hypergonadotropic hypogonadism. Journal of Clinical Endocrinology & Metabolism 1972.	321
51	Zorgniotti AW. Auto-injection of the corpus cavernosum with a vasoactive drug-combination for vasculogenic impotence. Journal of Urology 1985.	319
52	de Roux N. A family with hypogonadotropic hypogonadism and mutations in the gonadotropin-releasing hormone receptor. New England Journal of Medicine 1997.	314
53	Martin-Morales A. Prevalence and independent risk factors for erectile dysfunction in Spain: Results of the Epidemiologia de la Disfuncion Erectil Masculina study. Journal of Urology 2001.	308
54	Saleh RA. Oxidative stress and male infertility: From research bench to clinical practice. Journal of Andrology 2002.	307
55	Derby CA. Modifiable risk factors and erectile dysfunction: Can lifestyle changes modify risk? Urology 2000.	301
56	Linet OI. Efficacy and safety of intracavernosal alprostadil in men with erectile dysfunction. New England Journal of Medicine 1996.	300
57	Agarwal A. Clinical relevance of oxidative stress in male factor infertility: An update. American Journal of Reproductive Immunology 2008.	299
58	Lue TF. Vasculogenic impotence evaluated by high-resolution ultrasonography and pulsed doppler spectrum analysis. Radiology 1985.	298
59	Rosen RC. The multinational Men’s Attitudes to Life Events and Sexuality (MALES) study: I. Prevalence of erectile dysfunction and related health concerns in the general population. Current Medical Research and Opinion 2004.	297
60	Finkelstein JS. Osteoporosis in men with idiopathic hypogonadotropic hypogonadism. Annals of Internal Medicine 1987.	295
61	Kodama H. Increased oxidative deoxyribonucleic acid damage in the spermatozoa of infertile male patients. Fertility and Sterility 1997.	293
62	Lapatto R. Kiss1(−/−) mice exhibit more variable hypogonadism than Gpr54(−/−) mice. Endocrinology 2007.	291
63	Smith JC. The effects of induced hypogonadism on arterial stiffness, body composition, and metabolic parameters in males with prostate cancer. Journal of Clinical Endocrinology & Metabolism 2001.	288
64	McCulloch DK. The prevalence of diabetic impotence. Diabetologia 1980.	287
65	Debraekeleer M. Cytogenetic studies in male-infertility - a review. Human Reproduction 1991.	277
66	Selvin E. Prevalence and risk factors for erectile dysfunction in the US. American Journal of Medicine 2007.	276
67	Mulryan K. Reduced vas deferens contraction and male infertility in mice lacking P2X(1) receptors. Nature 2000.	275
68	Solomon H. Erectile dysfunction and the cardiovascular patient: endothelial dysfunction is the common denominator. Heart 2003.	270
69	Hendren SK. Prevalence of male and female sexual dysfunction is high following surgery for rectal cancer. Annals of Surgery 2005.	269
70	Finkelstein JS. Increases in bone-density during treatment of men with idiopathic hypogonadotropic hypogonadism. Journal of Clinical Endocrinology & Metabolism 1989.	269
71	Chang CS. Infertility with defective spermatogenesis and hypotestosteronemia in male mice lacking the androgen receptor in Sertoli cells. Proceedings of the National Academy of Sciences of the United States of America 2004.	258
72	Wang C. Investigation, treatment and monitoring of late-onset hypogonadism in males. European Journal of Endocrinology 2008.	256
73	Weiss J. Hypogonadism caused by a single amino-acid substitution in the beta subunit of luteinizing-hormone. New England Journal of Medicine 1992.	254
74	Kaiser DR. Impaired brachial artery endothelium-dependent and -independent vasodilation in men with erectile dysfunction and no other clinical cardiovascular disease. Journal of the American College of Cardiology 2004.	253
75	Ellenber M. Impotence in diabetes - neurologic factor. Annals of Internal Medicine 1971.	249
76	Althof SE. EDITS: Development of questionnaires for evaluating satisfaction with treatments for erectile dysfunction. Urology 1999.	247
77	Slag MF. Impotence in medical clinic outpatients. Journal of the American Medical Association 1983.	246
78	Kapoor D. Clinical and biochemical assessment of hypogonadism in men with type 2 diabetes: Correlations with bioavailable testosterone and visceral adiposity. Diabetes Care 2007.	244
79	Maden C. History of circumcision, medical conditions, and sexual-activity and risk of penile cancer. Journal of the National Cancer Institute 1993.	243
80	Palermo GD. Intracytoplasmic sperm injection - a novel treatment for all forms of male factor infertility. Fertility and Sterility 1995.	242
81	Woolf PD. Transient hypogonadotropic hypogonadism caused by critical illness. Journal of Clinical Endocrinology & Metabolism 1985.	242
82	Ghofrani HA. Sildenafil: from angina to erectile dysfunction to pulmonary hypertension and beyond. Nature Reviews Drug Discovery 2006.	240
83	Nicolosi A. Epidemiology of erectile dysfunction in four countries: Cross-national study of the prevalence and correlates of erectile dysfunction. Urology 2003.	240
84	Bivalacqua TJ. RhoA/Rho-kinase suppresses endothelial nitric oxide synthase in the penis: A mechanism for diabetes-associated erectile dysfunction. Proceedings of the National Academy of Sciences of the United States of America 2004.	239
85	de Kretser DM. Male infertility. Lancet 1997.	239
86	Macleod J. The male factor in fertility and infertility .2. Spermatozoon counts in 1000 men of known fertility and in 1000 cases of infertile marriage. Journal of Urology 1951.	239
87	Sharma RK. The reactive oxygen species - total antioxidant capacity score is a new measure of oxidative stress to predict male infertility. Human Reproduction 1999.	238
88	McVary KT. Sildenafil citrate improves erectile function and urinary symptoms in men with erectile dysfunction and lower urinary tract symptoms associated with benign prostatic hyperplasia: A randomized, double-blind trial. Journal of Urology 2007.	235
89	User HM. Penile weight and cell subtype specific changes in a post-radical prostatectomy model of erectile dysfunction. Journal of Urology 2003.	233
90	Seminara SB. Gonadotropin-releasing hormone deficiency in the human Idiopathic hypogonadotropic hypogonadism and Kallmann’s syndrome: Pathophysiological and genetic considerations. Endocrine Reviews 1998.	233
91	Korenman SG. Secondary hypogonadism in older men - its relation to impotence. Journal of Clinical Endocrinology & Metabolism 1990.	231
92	Talcott JA. Patient-reported impotence and incontinence after nerve-sparing radical prostatectomy. Journal of the National Cancer Institute 1997.	229
93	Montague DK. American Urological Association guideline on the management of priapism. Journal of Urology 2003.	228
94	Marks LS. Effect of testosterone replacement therapy on prostate tissue in men with late-onset hypogonadism - A randomized controlled trial. Journal of the American Medical Association 2006.	226
95	Benet AE. The epidemiology of erectile dysfunction. Urologic Clinics of North America 1995.	225
96	Sikka SC. Role of oxidative stress and antioxidants in male infertility. Journal of Andrology 1995.	225
97	Balhorn R. Aberrant protamine-1 protamine-2 ratios in sperm of infertile human males. Experientia 1988.	225
98	Daling JR. Penile cancer: importance of circumcision, human papillomavirus and smoking in in situ and invasive disease. International Journal of Cancer 2005.	224
99	Cummins JM. Molecular-biology of human male-infertility - links with aging, mitochondrial genetics, and oxidative stress. Molecular Reproduction and Development 1994.	224
100	Saleh RA. Negative effects of increased sperm DNA damage in relation to seminal oxidative stress in men with idiopathic and male factor infertility. Fertility and Sterility 2003.	218

**Fig. 1:**
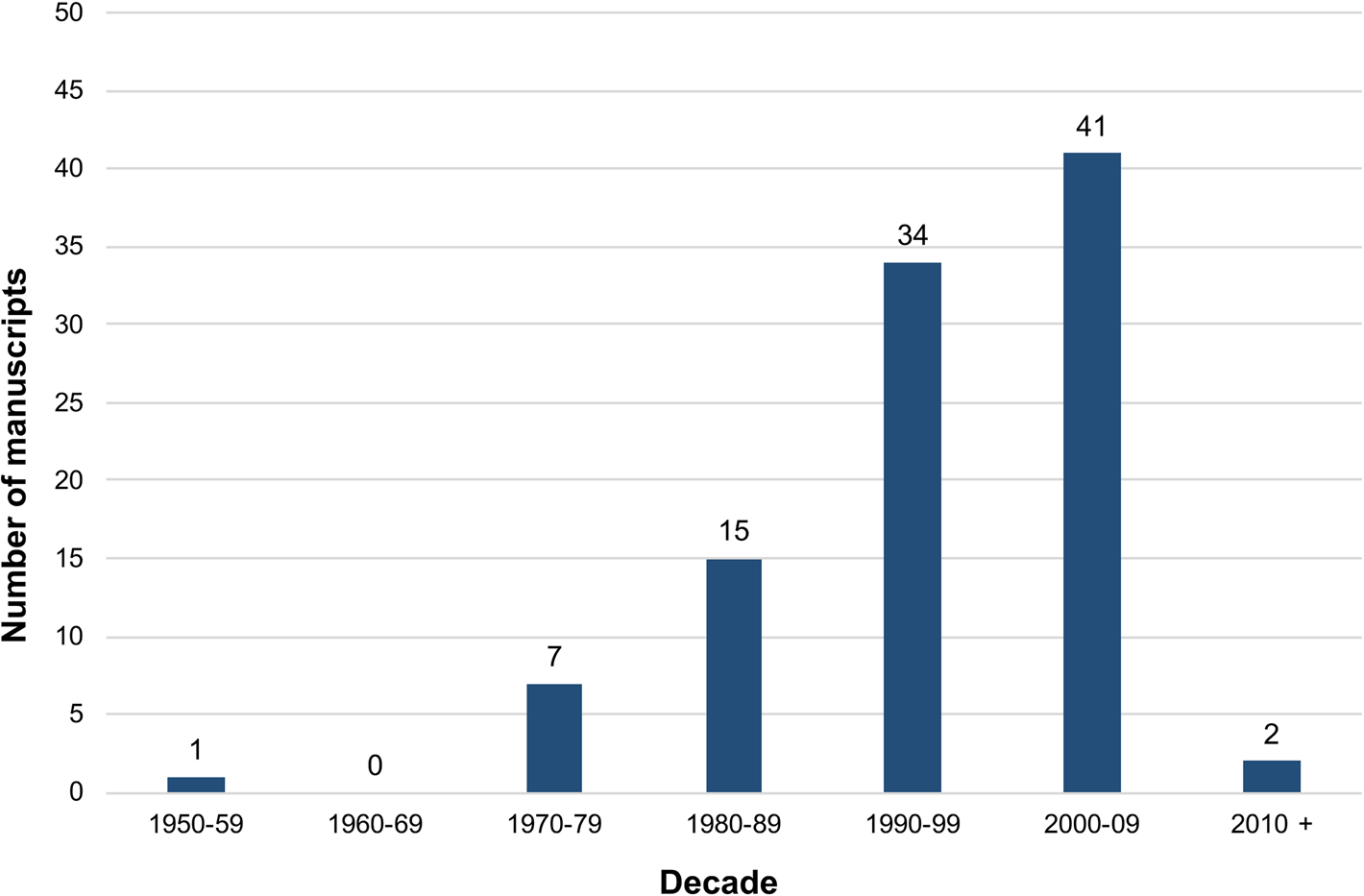
Bar graph demonstrating the distribution of the 100 most cited articles according to the decade in which they were published

The 100 most cited manuscripts were published over a broad time period, with the greatest proportion between 2000 and 2009 (*n* = 41), as demonstrated in Fig. 1. The most historic article was that by Macleod and Gold reporting comparative semen analysis in both ‘fertile’ and ‘infertile’ men, published in The Journal of Urology in 1951 and cited 239 times [14]. The most recent manuscript was that published in 2010 in European Urology by Hatzimouratidis, et al. outlining the European Association of Urology guidelines on investigation and management of male sexual dysfunction, which has been cited 420 times [15].

Table 2 outlines the 43 journals in which the top 100 manuscripts were published. The Journal of Urology (impact factor 5.157) published the highest number (*n* = 11), including the most cited article by Feldman et al. [13], and accrued a total overall citation number of 6771. This was followed by The New England Journal of Medicine, which published 10 manuscripts and was also the journal with the highest impact factor (72.406).

**Table 2:** Journals in which the 100 most cited manuscripts were published, ranked according to number with corresponding impact factors at the time of review

Journal Title	Impact Factor as of 2016	Number of Manuscripts in Top 100	Total number of citations
Journal of Urology	5.157	11	6771
New England Journal of Medicine	72.406	10	5388
Journal of Clinical Endocrinology and Metabolism	5.455	8	2610
Journal of the American Medical Association	44.405	6	3074
Urology	2.309	5	3784
Fertility and Sterility	4.447	4	1171
Proceedings of the National Academy of Sciences of the United States of America	9.661	4	2127
European Urology	16.265	3	1435
Human Reproduction Update	11.748	3	1198
International Journal of Impotence Research	1.293	3	2187
Journal of Andrology	2.473	3	1142
Lancet	47.831	3	993
Nature	40.137	3	1264
Annals of Internal Medicine	17.202	2	544
Endocrinology	4.286	2	898
Human Reproduction	5.02	2	515
Journal of the National Cancer Institute	13.757	2	472
American Journal of Medicine	5.55	1	276
American Journal of Reproductive Immunology	3.013	1	299
Annals of Surgery	8.98	1	269
Bioorganic & Medicinal Chemistry Letters	2.454	1	421
British Journal of Psychiatry	6.347	1	374
British Journal of Urology^a^	1.69	1	423
British Journal of Urology International	4.439	1	553
British Medical Journal	17.215	1	421
Current Medical Research and Opinion	2.757	1	297
Diabetes Care	11.857	1	244
Diabetologia	6.08	1	287
Endocrine Reviews	15.745	1	233
European Journal of Endocrinology	4.101	1	256
Experientia	2.072	1	225
Heart	6.059	1	270
International Journal of Cancer	6.513	1	224
International Journal of Clinical Practice	2.14	1	331
Journal of the American College of Cardiology	19.896	1	253
Molecular Reproduction and Development	2.316	1	224
Nature Genetics	27.959	1	395
Nature Reviews Drug Discovery	57	1	240
Preventive Medicine	3.434	1	431
Psychosomatic Medicine	3.863	1	345
Radiology	7.296	1	298
Science	37.205	1	492
Urologic Clinics of North America	2.22	1	225

^a^Impact Factor available for 2000 only

The country with the greatest number of publications was the United States of America (*n* = 66), followed by the United Kingdom (*n* = 12) and Canada (*n* = 5). The Massachusetts General Hospital was the institution with the greatest number of manuscripts (*n* = 7), followed jointly by the Cleveland Clinic and New England Research Institute (n = 6 respectively), all of which are based in the USA. RC Rosen [16–19] and TF Lue [20–22] were the first authors with the highest number of manuscripts in the top 100, achieving 4 and 3 respectively. A number of senior authors published more than one manuscript, with JB McKinlay achieving the greatest (*n* = 5), including the most cited article [13, 23–26].

**Fig. 2:**
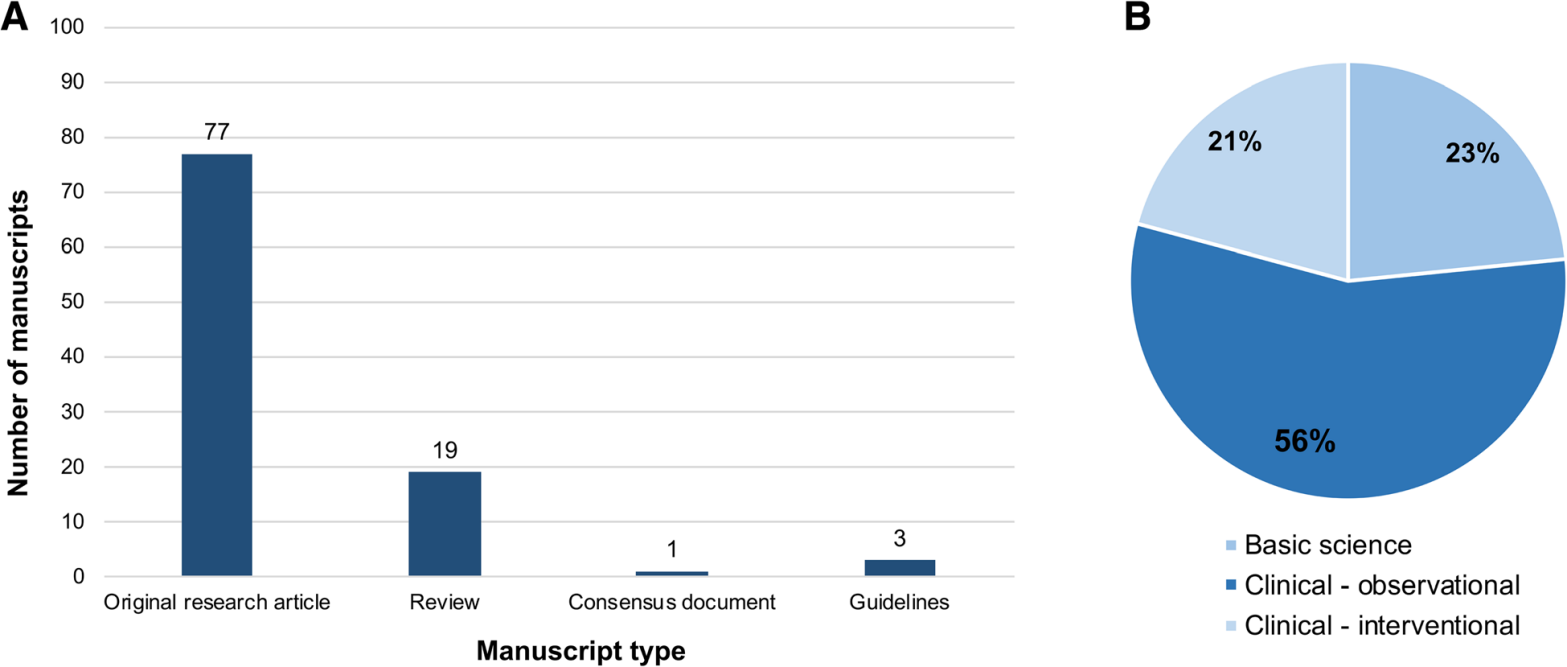
Manuscript type. **a** Bar graph demonstrating composition of the 100 most cited articles according to manuscript type. **b** Pie chart demonstrating the study design of the 77 original research articles

**Fig. 3:**
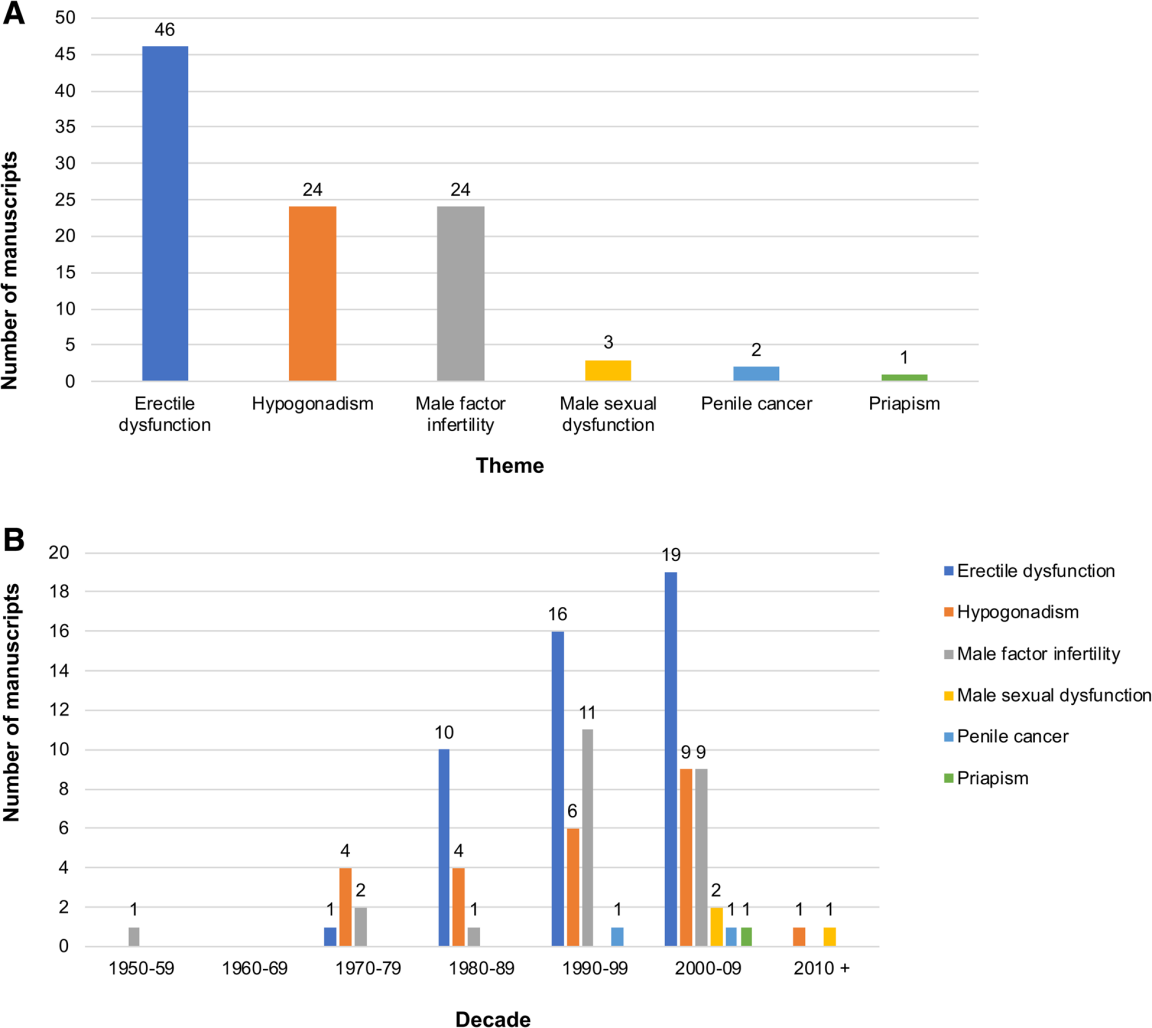
Bar graph demonstrating composition of the 100 most cited articles according to theme. **a** overall. **b** stratified according to decade of publication

Figure 2 gives the top 100 manuscripts according to type. The majority were original research articles (*n* = 77, Fig. 2a), of which 59 (76.6%) reported clinical outcomes (based on either observational or interventional methodology) and 18 (23.4%) reported the findings of basic scientific work (Fig. 2b). The number of manuscripts pertaining to each of the main andrology themes are given in Fig. 3a. ED was the most common (*n* = 46), followed jointly by hypogonadism and male factor infertility (*n* = 24 respectively). Figure 3b demonstrates manuscript theme as strafitied by decade of publication. Hypogonadism was the most common theme prior to 1980 (n = 4), after which ED remained the most common up until 2010. Despite an overall trend of increasing numbers of manuscripts focussing on ED within the top 100 during this period, the relative proportion of manuscripts fell with each decade (66.7, 47.1 and 46.3% between 1980 and 89, 1990–99 and 2000–09 respectivelty).

The citation rate of the top 10 manuscripts ranged from 118 to 45, as shown in Table 3. A number of articles from the top 10 remained the same when ranked by citation rate, although three were replaced by the more contemporary manuscripts by Wu, et al. (2010, 524 citations) [27], Hatzimouratidis, et al. (2010, 420 citations) [15] and Tremellen (2008, 479 citations) [28].

**Table 3:** Top 10 manuscripts with the highest citation rate

Rank	Citation Rate	First Author	Senior Author	Title	First author institution	Country
1	118	Rosen RC	Mishra A	The international index of erectile function (IIEF): A multidimensional scale for assessment of erectile dysfunction	University of Medicine and Dentistry of New Jersey	USA
2	117	Feldman HA	McKinlay JB	Impotence and its medical and psychosocial correlates - results of the Massachusetts male aging study	New England Research Institute	USA
3	82	de Roux N	Milgrom E	Hypogonadotropic hypogonadism due to loss of function of the KiSS1-derived peptide receptor GPR54	Hôpital de Bicêtre	France
4	76	Goldstein I	Wicker PA	Oral sildenafil in the treatment of erectile dysfunction	Boston University Medical Centre	USA
5	70	Rosen RC	Pena BM	Development and evaluation of an abridged, 5-item version of the International Index of Erectile Function (IIEF-5) as a diagnostic tool for erectile dysfunction	Robert Wood Johnson Medical School	USA
6	66	Wu FCW	Huhtaniemi IT	Identification of Late-Onset Hypogonadism in Middle-Aged and Elderly Men	University of Manchester	UK
7	53	Hatzimouratidis K	Wespes E	Guidelines on Male Sexual Dysfunction: Erectile Dysfunction and Premature Ejaculation	Aristotle University of Thessaloniki	Greece
8	51	Droller MJ	Hall WH	Impotence: NIH consensus development panel on impotence	Mount Sinai Medical Center	USA
9	48	Tremellen K	Tremellen K	Oxidative stress and male infertility - a clinical perspective	Repromed & University of Adelaide	Australia
10	45	Rosen RC	Giuliano F	Lower urinary tract symptoms and male sexual dysfunction: The multinational survey of the aging male (MSAM-7)	Robert Wood Johnson Medical School	USA

## Discussion

This study is the first bibliometric analysis to identify and analyse the most influential manuscripts in the field of andrology. A range of topics were represented within the top 100, which reflects the spectrum of clinical andrological practice as well as the variation in the specialist backgrounds of andrological practitioners and researchers. Similarly, there is significant overlap with other medical specialties including, but not limited to, endocrinology. Despite this overlap, the most prevalent theme of publication was the assessment and/or treatment of patients with ED, constituting 46 papers within the top 100. This included the most cited article by Feldman, et al. [13] describing the prevalence of, and risk factors for, ED in the Massachusetts Male Ageing Study, published in The Journal of Urology in 1994 and cited 2819 times. The Massachusetts Male Aging Study was a community-based, observational survey of non-institutionalised men between 40 and 70 years old conducted between 1987 to 1989 in cities and towns near Boston, Massachusetts. The aim was to correlate a self-administered erectile function questionnaire with an assessment of patients’ overall health. The authors noted a strong correlation with patients who had vascular, cardiac or smoking-related diseases and concluded that ED was associated with potentially reversible age-related changes.

The self-administered erectile function questionnaire reported in the Feldman, et al. study differed from the now more commonly used International Index of Erectile Function (IIEF), which was first described three years later in 1997 by Rosen, et al. [16]. This landmark publication constituted the second most cited article in this bibliometric analysis. Similarly, the fourth most cited article described an updated version of the IIEF questionnaire and was again published by Rosen, et al. [17]. The reason that these papers have been cited so frequently is most likely due to both the high overall prevalence of publications focussing on ED, and the fact that the IIEF-5 is widely used in clinical practice to measure the severity of ED and is hence frequently used in research studies as an ‘objective’ measurement of function, treatment efficacy or response.

Fourteen manuscripts in the top 100 focussed specifically on the treatment of ED. These ranged in age from the article by Brindley in 1983 examining the effects of intra-cavernosal alpha-blockade [29], to that by McVary, et al. in 2007 reporting the results of a randomised controlled trial investigating the effects of oral sildenafil on both ED and urinary symptoms [30]. This analysis demonstrates that there was a significant increase in the number of influential manuscripts focussing on the treatment of ED following publication of the landmark paper by Goldstein, et al. in 1998 reporting the effectiveness of oral sildenafil [31]. This was the first paper to describe an oral treatment for ED and is the third most cited manuscript in the top 100. Prior to this time, treatments had been relatively intolerable for patients, including vacuum tumescence devices, intracavernosal injections of vasoactive agents, transurethral delivery of alprostadil, and invasive surgical procedures. The availability of oral treatments for ED subsequently resulted in a rapid increase in research being undertaken into all aspects of the condition, which is reflected in the numerous manuscripts published after 1998 that feature in the top 100 (Figs. 1 and 3b). Furthermore, it is probable that there was an increase in self-reporting of ED and a drive towards increased research funding and activity when a number of ‘tolerable’ treatments became available.

A significant majority of manuscripts were published by authors in the United States of America (*n* = 66), followed by the United Kingdom (*n* = 12) and Canada (*n* = 5). This disparity between the USA and other countries may reflect the subspecialisation that occurs in contemporary American Urological practice compared with other geographical regions [32]. This means that clinicians are more likely to be solely practicing andrology, with less emphasis on provision of ‘core urological’ services, therefore potentially allowing more time for academic pursuits. This geographical dominance has also been observed in other bibliometric analyses [7] and may also be explained by differences in academic focus in relation to clinical practice, with more funding provided to clinicians’ academic work in the USA compared to elsewhere, which in turn appears to correlate with a higher quality of research [33].

It is important to note that only two manuscripts within the top 100 focus on the management of penile cancer (Maden, ranked 79 [34] and Daling, ranked 98 [35]). This is the only cancer that is commonly managed by andrologists and therefore one would expect to see a greater number of manuscripts related to its pathophysiology, treatment and follow-up within the top 100. However, it is likely that the low incidence of this condition makes it a relative ‘Cinderella’ subspecialty within the field and therefore papers focussed on penile cancer are less widely cited when compared to the much more commonly encountered conditions of ED and hypogonadism [36]. Moreover, due to the paucity of cases seen in routine practice it is difficult to establish a cohort of sufficient size for high quality observational or interventional research. This, coupled with the known difficulties in conducting high quality surgical trials [37], means that manuscripts are often of low levels of evidence, thus precluding them from publication in high impact factor journals.

The journals in which the top 100 manuscripts were published varied significantly both in theme and impact factor. The latter was particularly varied and ranged from 72.406 to 1.293 (median 5.5025). Interestingly, impact factor did not necessarily correlate with the most cited papers. For example, the second most cited paper by Rosen, et al. [16] was published in ‘Urology’, which was the sixth-lowest ranked journal in terms of impact factor within this analysis (2.309). One explanation for this variation is the multidisciplinary nature of many conditions encountered under the umbrella of andrology. Furthermore, the subspecialised nature of many andrological conditions means that manuscripts pertaining to these topics are often not directly relevant to ‘core’ clinical practice and are therefore less likely to be published in higher impact factor journals that seek to meet the interests of a broad audience.

The main limitation of bibliometric analysis is the potential for a number of types of bias. Firstly, disproportionate citation may result from institutional bias, language biases, self-citation or powerful person bias. In addition, older manuscripts may receive more citations due to the length of time in which they are in the public domain. Although the use of citation rate attempts to control for this, it may take a number of years for influential manuscripts to accrue citations due to the publication lead-time for their citing manuscript. A further limitation is the inclusion of only first and senior authors, and the institution of the first author. It is possible that several first authors will have co-authored other papers in the top 100 and therefore be underrepresented in the current study. Finally, searching based on title means a small number of manuscripts that have key andrological themes without pertaining to these in their title may not have been identified.

## Conclusion

This list of highly cited papers identifies the topics and authors that have made the most impact in the discipline of andrology over the last century. There is a clear predominance of manuscripts focusing on the treatment and pathophysiology of ED, which should therefore be considered the most widely researched, published and cited field within andrological practice. This study provides a reference of what may be considered as the most influential papers in andrology and serves as a inidcation of what comprises a ‘highly citable’ manuscript for both researchers and clinicians.

## Abbreviations

ED: Erectile dysfunction
IIEF: International Index of Erectile Function
USA: United States of America
